# Transvaginal natural orifice transluminal endoscopic surgery (vNOTES) hysterectomy for uterus weighing ≥1 kg

**DOI:** 10.1186/s12893-020-00897-3

**Published:** 2020-10-12

**Authors:** Xiaojuan Wang, Junwei Li, Keqin Hua, Yisong Chen

**Affiliations:** Department of Gynecology, Obstetrics and Gynecology Hospital of Fudan University, 128 Shenyang Road, Shanghai, 200090 China

**Keywords:** Transvaginal natural orifice transluminal endoscopic surgery, Hysterectomy, Large uterus

## Abstract

**Background:**

The transvaginal natural orifice transluminal endoscopic surgery (vNOTES) applied in gynecology has been developed recent years and been evolving. In this study, we aimed to evaluate the feasibility and effect of the vNOTES hysterectomy for uterus ≥1 kilogram (kg).

**Methods:**

From January 2019 to March 2020, patients with benign indications in cases of uterus weighing ≥1 kg, underwent vNOTES hysterectomy were studied retrospectively. The patients’ demographics, indications for surgery, operation outcomes and follow-up details were recorded.

**Results:**

39 patients were performed vNOTES hysterectomy for large uterus (mean weight 1141.8 gram, range from 1000 to 1720), indications for surgery included bulky uterine myomas or adenomysosis. The mean age was 48 years (range 42–66) and mean BMI was 24 kg/m^2^ (range 18.4–38). Mean operating time was 123.3 min (rang 40–400) and the mean estimated blood loss was 206.7 milliliters  (range 10–1300). The mean pain assessment was 2.1 (range 0–5). The mean length of stay was 2.4 nights (1–11). 1 patient experienced ureteral injury and was performed ureteral anastomosis. 3 patients were converted to vaginal-assisted trans-umbilicus single-port laparoscopy. The learning curve was analyzed to show that 20 cases were needed to achieve proficiency in vNOTES hysterectomy for large uterus ≥1 kg.

**Conclusion:**

Our preliminary experience suggested that vNOTES hysterectomy for large uterus weighing ≥1 kg was feasible and safe, meanwhile this procedure had the advantages of all the minimal invasive approach such as fast recovery and aesthetic advantage.

## Background

Natural orifice transluminal endoscopic surgery (NOTES) is an emerging technique, and the transvaginal route is the most common approach [1] . Transvaginal NOTES (vNOTES) has attracted attention as a less invasive procedure, has the better cosmetic results than conventional laparoscopy, decreased postoperative pain, reduced hospital stays and recovery as well as decreased perioperative morbidity [2].

The previous publications have confirmed vNOTES to be a safe and feasible procedure for hysterectomy and adenextomy [3, 4]. However, there were no literatures reported about vNOTES hysterectomy in large uterus, especially in uterus weighing ≥1 kg (kg). Not only the open abdominal surgery, but also the traditional laparoscopy, removing a very large uterus may be extremely difficult, due to the problem in the mobilization of the uterus and poor visualization of the pelvic structures [5]. The aim of our study was to evaluate the feasibility and effect of the vNOTES hysterectomy procedure in large uterus weighing ≥1 kg and to access the learning curve based on operative times and short-term outcomes.

## Methods

We reviewed the medical charts and all consecutive cases of vNOTES hysterectomy for benign indications with uterus weighing ≥1 kg performed by a single surgeon (Y.S. C) at the Department of Gynecology, Obstetrics and Gynecology Hospital of Fudan University, Shanghai, China, between January 2019 and March 2020. The baseline demographic data were recorded, including age, parity, body mass index (BMI), comorbidity, and history of surgery. The perioperative outcomes, such as operative time, estimated blood loss, uterine weight, changes in hemoglobin levels, hospital stay, any peri- or postoperative complications (fever, bowel injury, or genitourinary tract injury), and final pathologic diagnosis were recorded. The change in hemoglobin was calculated as the preoperative hemoglobin value minus the hemoglobin value on the first day after surgery. The length of hospital stay was calculated from the first day after surgery. Organ damage was considered as intraoperative complication. This study was approved by the Institutional Review Board and Ethics Committee (Number 2019–32) of Obstetrics and Gynecology Hospital of Fudan University.

All women were performed a routine preoperative assessment including a bimanual pelvic examination or vagino-recto-abdominal examination, a Papanicolaou smear, magnetic resonance imaging, and urinary ultrasound. Women with abnormal uterine bleeding were given diagnostic hysteroscopy and endometrial biopsy. All patients were given the written informed consent to the surgical procedure and to the use of individual data for research.

### Surgical techniques

**Fig. 1:**
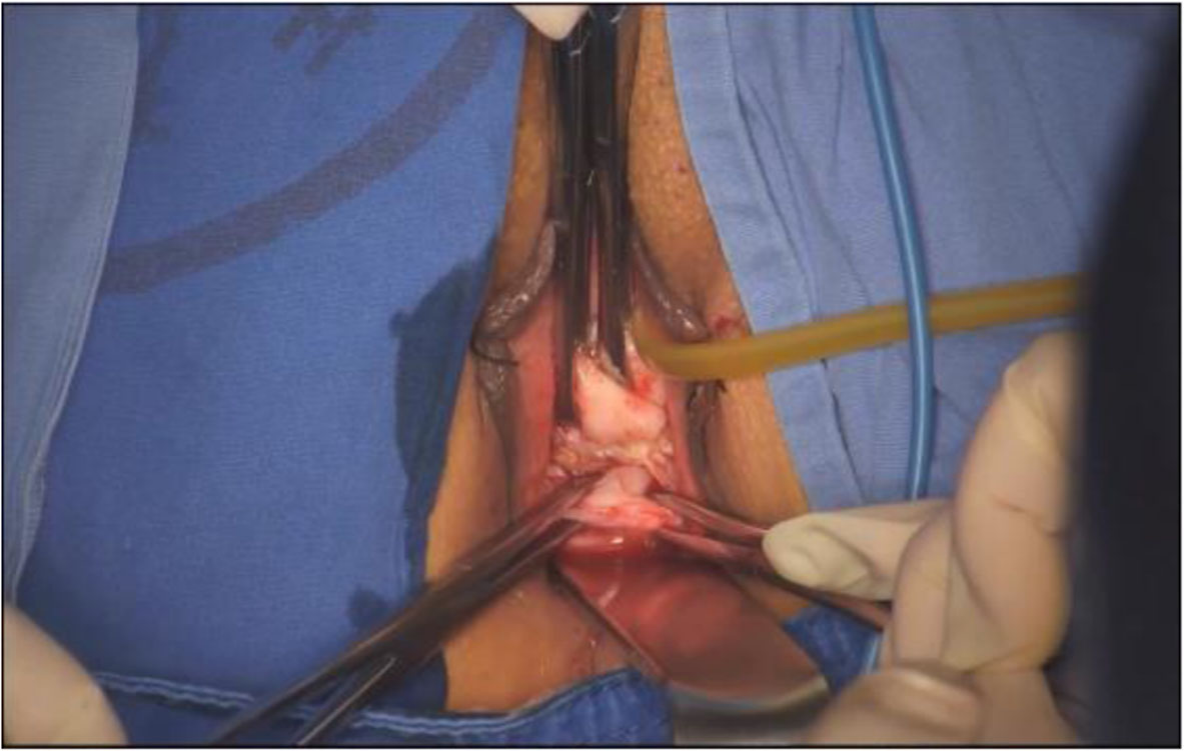
The rectovaginal spaces was opened

**Fig. 2:**
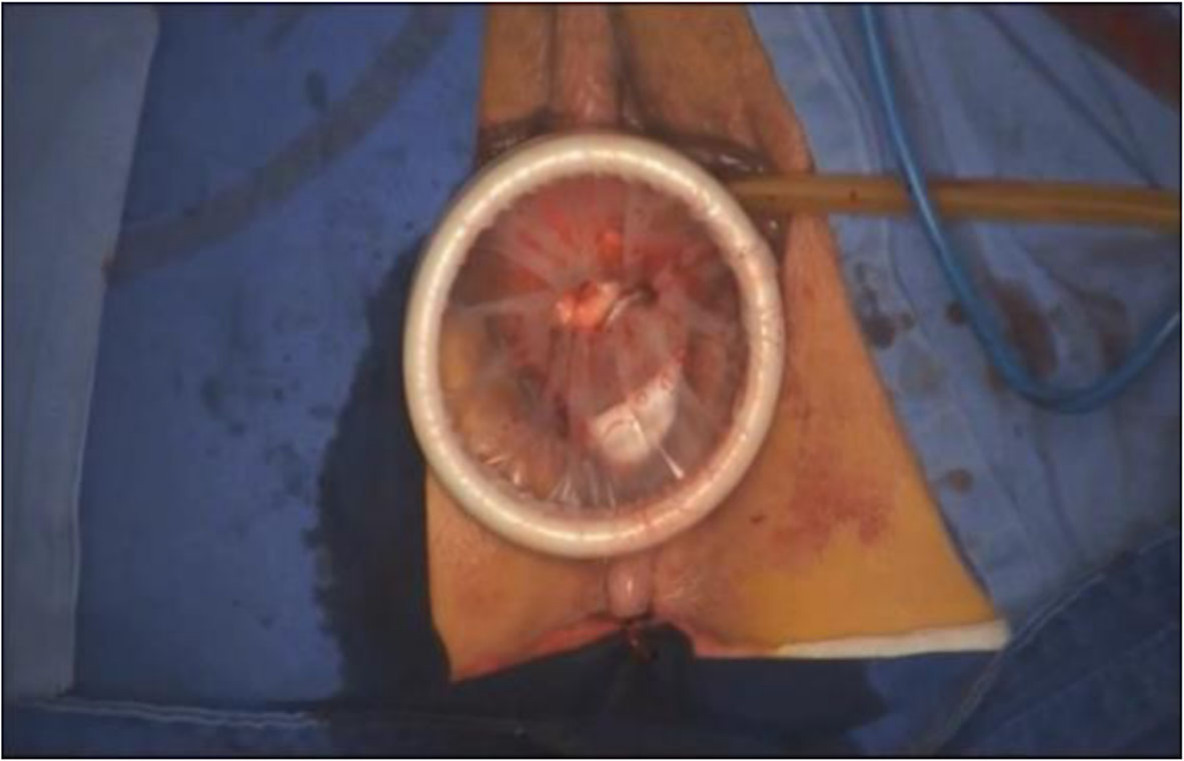
The single- ports device was established

Patients were administered general anesthesia and placed in the dorsal lithotomy, and a Foley catheter was then inserted for constant urinary drainage. After injection of a water cushion, the circumcision of the vaginal mucosa around the cervix was performed, and the posterior portion was carried out by pushing up the vaginal mucosa along with the uterine-cervical fascia at the posterior fornix, then the rectovaginal space was opened (Fig. 1). The posterior colpotomy was easily underwent, however, sometimes the anterior colpotomy was not completed and was performed during the laparoscopy. The bilateral board ligaments, the transverse cervical and the uterosacral ligament complexes were coagulated and cut, then sutured. Then the single- ports device (Hangtian technology, China) was established (Fig. 2). The endoscope we used was a 10-mm, 30-degree endoscope (Karl Storz GmbH & Co KG, Tuttlingen, Germany).

**Fig. 3:**
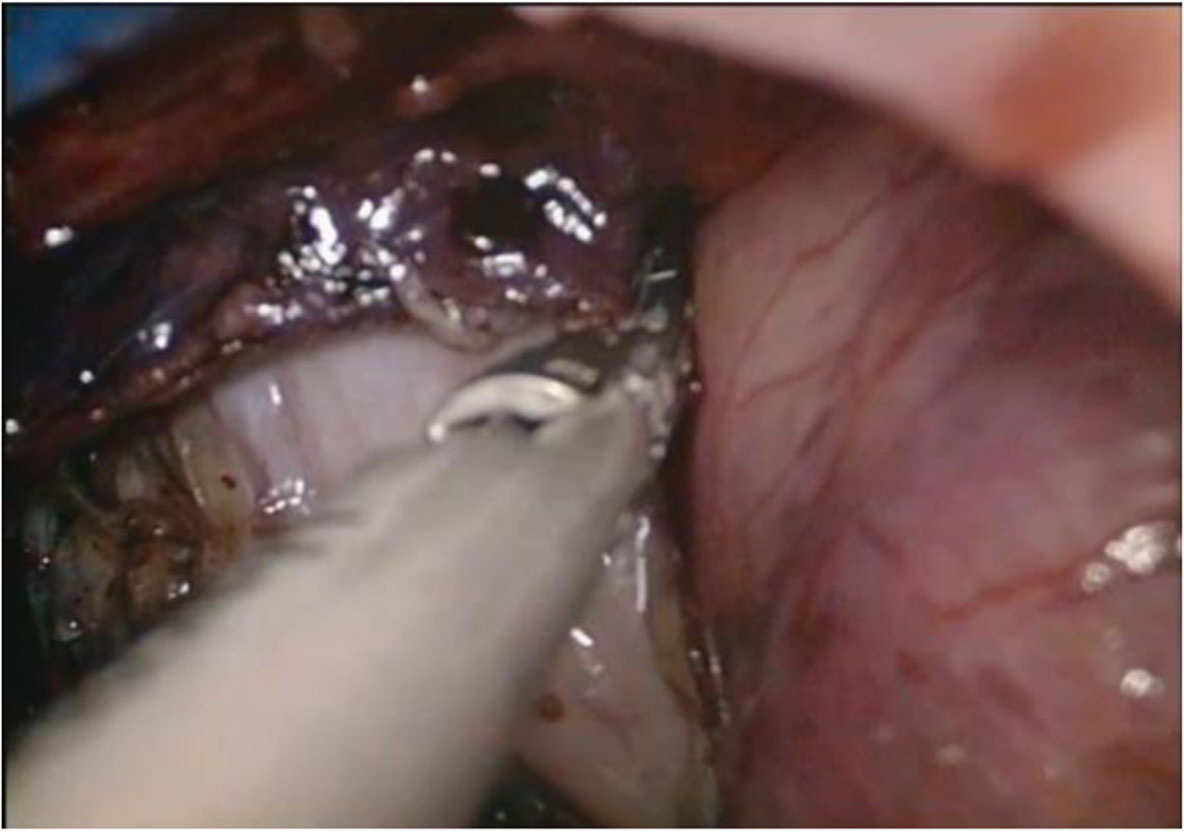
The uterovesical junction was traced and identified

**Fig. 4:**
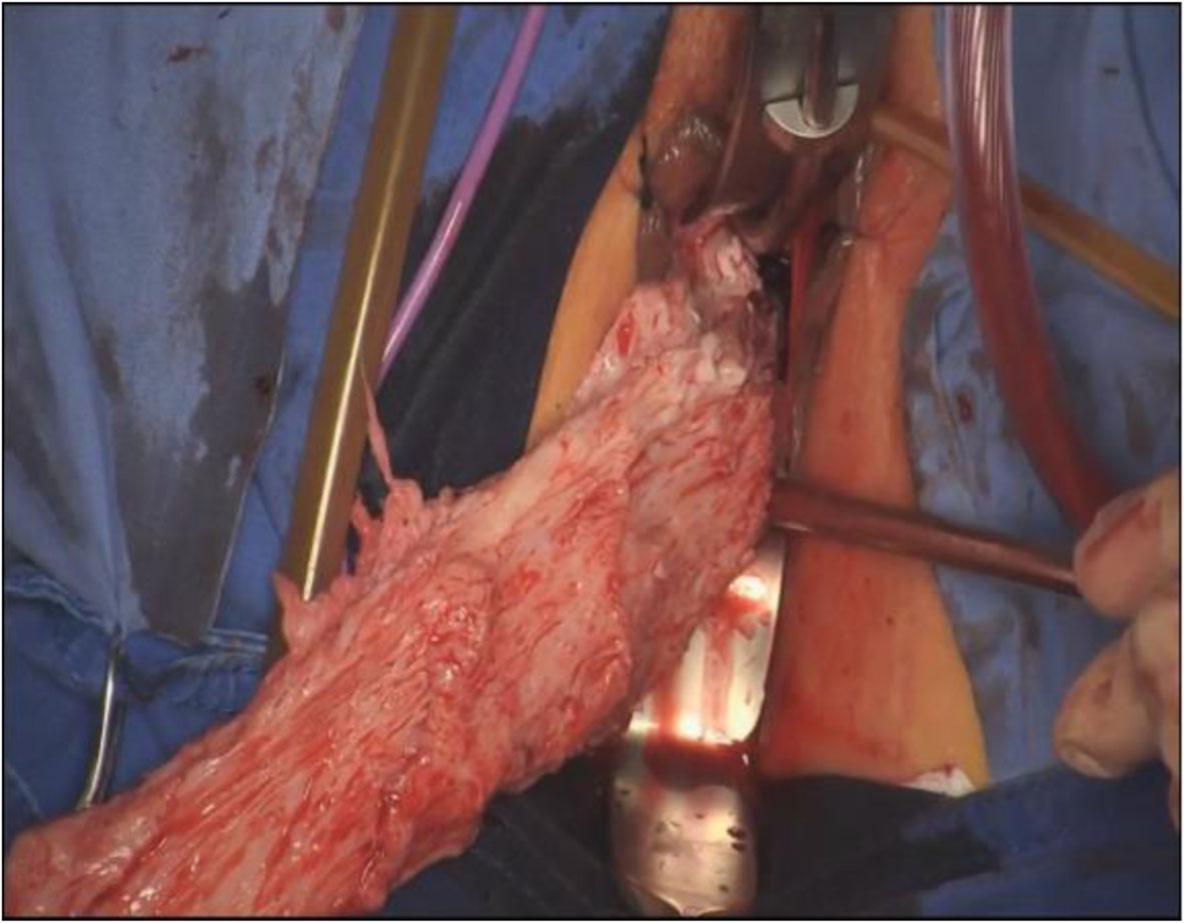
Extracting the uterus was performed using a cold knife transvaginally


After the creation of pneumoperitoneum, the bilateral broad ligaments of the uterine vessels were ascertained by grasping the cervix and pushing toward the contralateral site with endoscopic allis clamp. The uterine vessels were fixed and cut by a 5-mm bipolar Ligasure vessel sealer (Covidien, Mansfield, MA). Following the stump of the uterine arteries and the anterior border of uterus, the uterovesical junction was traced and identified (Fig. 3). After dissecting the uterovesical junction with ultrasonic knife, the anterior colpotomy was completed under laparoscopic guidance. If there were pelvic adhesions, ultrasonic knife or scissors was used for decompose adhesion. The remaining board and round ligaments were grasped and cut off step by step using Ligasure. The uterus was placed inside a large endobag, specimen was cut rotatingly by a cold knife, and extraction of the uterus was accomplished transvaginally (Fig. 4). Finally, the peritoneum and vaginal vault were closed with Vicryl suture (Vicryl; Ethicon Inc.). Pelvic floor reconstruction was performed in some cases. The surgical procedures were seen in the supplementary information.

**Additional file 1**

Antibiotic prophylaxis was administered 0.5 h before surgery; antithrombotic prophylaxis was given in accordance with the Caprini deep vein thrombosis assessment [6]. The Foley catheter was removed on the first day after the operation. The patients were discharged, if their vital signs were stable and there was no evidence of surgical complications. Patients were required to forbid sex activity for at least 6 weeks after the operation and to return the outpatient 1 month after operation for follow-up examinations.

### Statistical analysis

Descriptive statistics were expressed in terms of quantitative value as mean standard deviation (SD) or median and range and percentage. Linear regression was conducted to assess the sign of the slope of the regression for the learning curve. Independent t-tests were used to compare the continuous variables. A *p*-value less than 0.05 was considered statistically significant. SPSS software (SPSS version 22.0; SPSS Inc., Chicago, IL) was used to calculate.

## Results

During the study period, a total of 39 patients underwent vNOTES hysterectomy for uteri weighing ≥1 kg with no suspected preoperative malignancy. The indications for surgery included 20 cases of bulky uterine myomas and 19 cases of adenomyosis, and the final histological examination revealed no unexpected malignant diseases. Menorrhagia represented at 5 patients. The detailed patient characteristics were seen in Table 1. Mean age was 48 years (range 42–66); the mean BMI was 24 kg / m^2^ (range 18.4–38); 3 patients (7.7%) were nulliparity, and 32 patients (82.1%) were vaginal births. 9 patients (23.1%) experienced myomectomy or cesarean section, and 7 patients (18%) experienced other abdominal surgeries. 12 patients (30.8%) were anemia preoperatively, and the mean preoperative hemoglobin was 106 g/dL (range 74–138).

**Table 1:** Demographic characteristics of the Patients

Characteristic	Value
Age in years, mean (range)	48 (42–66)
BMI, kg/m^2^, mean (range)	24 (18.4–38)
Nulliparity, number (%)	3 (7.7%)
Vaginal births, number (%)	32 (82.1%)
Hyspertension, number (%)	4 (10.3%)
Previous surgery on the uterus (CS, mymoectomy), number (%)	9 (23.1%)
Previous abdominal surgery (excluding CS or mymectomy), number (%)	7 (18%)
Preoperative anemia (Hb < 10.5 g/dL), number (%)	12 (30.8%)
Preoperative hemoglobin (g/dL), mean (range)	106 (74–138)

*BMI* body mass index; *CS* cesarean section; *HB* hemoglobin

Mean operating time was 123.3 min (rang 40–400), mean estimated blood loss was 206.7 milliliters (range 10–1300). 2 patients (5.1%) were given blood transfusion. There were 8 patients (20.5%) being underwent with bilateral sapingo-oopherectomy concomitantly, 25 patients (64%) with bilateral salpingectomy, 2 patients (5.1%) with pelvic floor reconstruction, 13 patients (33.3%) with pelvic adhesion decomposition, and 2 patients (5.1%) with ovarian cystectomy. The mean uterus weight was 1141.8 gram (g) (range 1000–1720). The mean pain assessment was 2.1 (range 0–5). The mean length of stay was 2.4 nights (1–11). There was 1 patient experienced ureteral injury, who was performed ureteral anastomosis. 3 patients were converted to vaginal-assisted trans-umbilicus single-port laparoscopy. The perioperative details were seen in Table 2.

**Table 2:** Perioperative Details

Characteristics	Values
Operation time, minutes mean (range)	123.2 (40–435)
Estimated Blood loss, milliliters mean (range)	206.7 (10–1300)
Blood transfusion, number (%)	2 (5.1%)
**Concomitant surgeries**	
Bilateral sapingo-oopherectomy, number (%)	8 (20.5%)
Bilateral salpingectomy, number (%)	25 (64%)
Pelvic floor reconstruction, number (%)	2 (5.1%)
Pelvic adhesion decomposition, number (%)	13 (33.3%)
Ovarian cystectomy, number (%)	2 (5.1%)
Uterus weight (g), mean (range)	1141.8 (1000–1720)
Pain assessment (VAS: 0–10), mean (range)	2.1 (0–5)
Hb drop (g/dL), mean (range)	0.1 (0–0.4)
Length of hospital stay, nights mean (range)	2.4 (1–11)
Intraoperative complications, number (%)	1 (2.6%)
Conversions, number (%)	3 (7.7%)

Excluding 2 patients with concomitant pelvic floor reconstruction, 3 patients with conversion of vaginal-assisted single-port laparoscopy and 1 patient with complication, we compared the first 20 patients and the sequential 13 procedures, and found mean operative time decreased from 121.4 min (± 39.8) in the first 20 patients to 81.6 min (± 23.4) in the 13 patients greatly (*p* = 0.001). In addition, The estimated blood loss was decreased from 168.5 milliters (± 173.5) in the first 20 patients to 76.1 milliliters (± 55.5) in the sequential 13 patients significantly (*p* = 0.037); and the length of hospital stay decreased from 2.25 nights (± 0.85) in the first 20 patients to 1.61 nights (± 0.65) in the sequential 13 patients (*p* = 0.022). There was no difference of Hb drop in the first 20 patients and the sequential 13 patients. The above was seen in Table 3. And there was no patient associated with any complications during one-month follow up after operation.

**Table 3:**
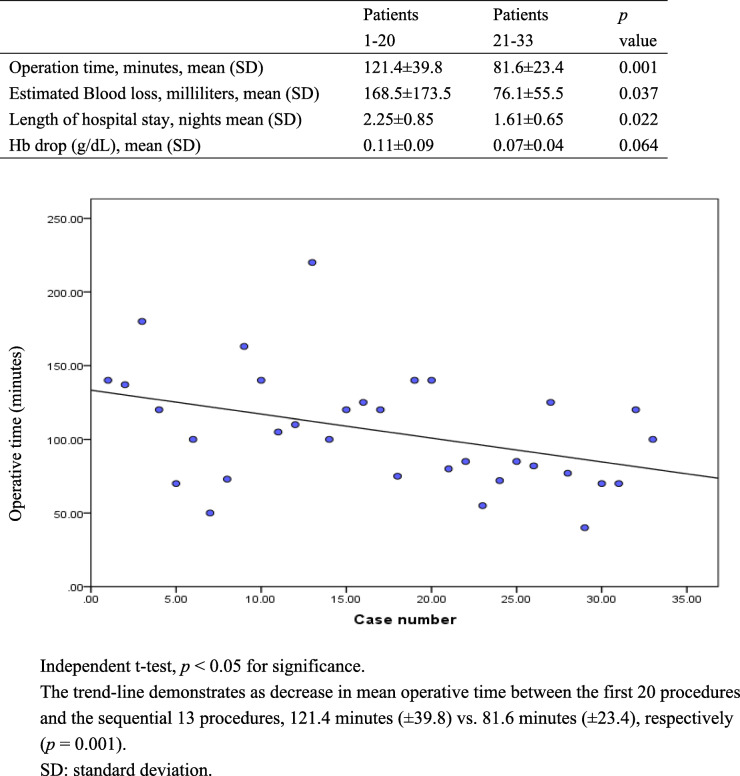
Learning curve

## Discussion

Hysterectomy is the most common surgical procedure in the domain of gynecology, and there are several surgical approaches to hysterectomy: abdominal hysterectomy (AH), vaginal hysterectomy (VH), laparoscopic hysterectomy (LH) with multi-port or single-port, robotic-assisted hysterectomy, and the emerging vNOTES hysterectomy [3, 7]. Several randomized trials and meta-analyses had shown that the minimally invasive approach (VH or LH) was clearly superior to AH in terms of perioperative outcomes and recovery time [8, 9], and recommend VH as a feasible procedure for the patients with benign indication and the LH as an alternative approach with the increased risk of urinary tract injury [9]. However, there were some difficulties of the mobilization of the uterus and poor visualization of the pelvic structure during the LH, and the published literatures reported that LH in large uterus was feasible and safe [5, 10].

The vNOTES combined conventional vaginal surgery and single-port laparoscopy as an emerging technique, several studies revealed that vNOTES hysterectomy for benign indications could be a feasible and safe approach, and had advantages over traditional laparoscopic and vaginal hysterectomy, such as low complications, minimal post-surgical pain, fast recovery and short hospitalization [3, 11–13]. Currently, there was no publication about vNOTES hysterectomy in large uterus. Compared to VH, vNOTES hysterectomy could better access and the ureters can be easy to identify. And compared to the LH, regardless of scarless per abdomen and trocar related injuries, the pelvic space could be improved by pulling the uteri towards the head during vNOTES hysterectomy in large uterus.

In our study, the mean uterus weight was 1141.8 g (range 1000–1720), which was kept in accordance with the publications of LH [5, 10, 14–16]. In LH for large uterus, there were several cases which converted to AH, due to dense adhesions, narrow vaginal access, intraoperative hemorrhage, bulky myomas’ location. In our study, there were 3 (7.7%) women converted from an initially intended vNOTES to vaginal-assisted trans-umbilicus single-port laparoscopy, the major reason for conversion was being unable to posterior colpotomy; two of them had history of myomectomy, one of them was nulliparity. Even though the conversion rate (7.7%) was higher that of LH (5.2%) [16], we just converted vaginal-assisted trans-umbilicus single-port laparoscopy, which also belonged to minimally invasive approach. One of 39 procedures (2.6%) experienced intraoperative complication—right ureter injury, this was a nulliparity patient with endometriosis with adenomyoma, who was associated with serious pelvic adhesion. And this patient had stay hospital for 11 nights, which was greater than the mean 2.4 nights. Therefore, it was necessary for the selected conditions for vNOTES hysterectomy for large uterus.

Regardless of the cases of conversion and concomitant pelvic floor reconstruction, we found that the significant improvement the learning curve between the first 20 procedures and the sequential 13 procedures, which manifested in a reduction of one-third of the operative time for vNOTES, nearly one-half reduction of estimated blood loss, as well as a significant reduction of hospital stay. It’s reported that the learning curve of vNOTES hysterectomy was greatly improved after 10 procedures [17], however, the volume of uterus was not included.

To our knowledge, this is the first article to describe the surgical experience with vNOTES hysterectomies for uterus weighing ≥1 kg, even in the morbid obesity (BMI = 38). We also recognize some limitations; the limited sample may not reflect the true incidence of surgical complications; this is an observational study with no comparative arm; and all the procedures were performed by an experienced surgeon who is proficient about the vaginal and single-port laparoscopic surgery technique. That is, the outcomes may not be suitable for all the gynecologic public. However, because surgical skills can be taught and improved, we believe that more and more gynecologic surgeon should accomplish such difficult cases.

## Conclusions

Our preliminary experienced suggested that the vNOTES hysterectomy for large uteri weighing ≥1 kg was feasible and safe, meanwhile this procedure had the advantages of all the minimally invasive approach such as fast recovery and aesthetic advantage.

## Data Availability

The datasets used and analyzed during the current study available from the corresponding author on reasonable request.

## Abbreviations

vNOTES: transvaginal natural orifice transluminal endoscopic surgery
kg: kilogram
NOTES: Natural orifice transluminal endoscopic surgery
g: gram
AH: Abdominal hysterectomy
VH: Vaginal hysterectomy
LH: Laparoscopic hysterectomy
